# Supermarket health advocacy, resources, and education: feasibility of a supermarket-based prediabetes and diabetes screening and education program

**DOI:** 10.3389/fpubh.2025.1582710

**Published:** 2025-06-18

**Authors:** Renee Cadzow, Andy Strohmeier, Jamie Keller, Ashley Regling, Marchelle Brooks, Teresa Quattrin

**Affiliations:** ^1^Clinical and Translational Science Institute, Jacobs School of Medicine and Biomedical Sciences, University at Buffalo, Buffalo, NY, United States; ^2^Division of Health Services Policy and Practice, Department of Epidemiology and Environmental Health, School of Public Health and Health Professions, University at Buffalo, Buffalo, NY, United States; ^3^Department of Pediatrics, Jacobs School of Medicine and Biomedical Sciences, University at Buffalo, Buffalo, NY, United States; ^4^Northeast Shared Services, Tops Markets, Buffalo, NY, United States

**Keywords:** diabetes prevention, diabetes management, supermarket intervention, community based, popular education

## Abstract

**Introduction:**

This project aimed to determine the feasibility of engaging supermarket patrons in diabetes screening, healthy food promotion and education to bridge geographic, economic, and knowledge gaps in diabetes prevention and management.

**Methods:**

Trained staff tabled at supermarket entrances advertising screening for pre-diabetes and diabetes. Customers without a diabetes diagnosis completed a National Diabetes Prevention Program Prediabetes Risk Test (score >5 = prediabetes risk). Those with a previous diabetes diagnosis completed a brief questionnaire on their diabetes knowledge/management, healthcare access, and social determinants of health. Surveys took about 5 minutes to complete. Participants received a $5 voucher for fruit and vegetables, evidence based educational material and a list of healthcare resources in the community. The results of the survey informed the design and implementation of 5 educational sessions using an adult learning, popular education approach. A $10 grocery voucher was given for attendance at each session.

**Results:**

303 customers of four grocery stores in urban Buffalo took the survey between January and June 2024. 67% of those screened were either at-risk for or were already diagnosed with diabetes. 227 people completed the Prediabetes Risk Test: 58% had a score >5 (indicating they were at risk for pre-diabetes), 51% reported having hypertension, and 75% reported a BMI categorized as overweight or obese. 76 participants (25%) stated they had been diagnosed with diabetes. Of these, 91% saw a doctor every 3 months, but 28% did not know the importance of HbA1c, 18% had trouble paying for medications, and 15% had inadequate transportation. 55 people (34 unique) participated in five educational sessions. Participants shared questions, concerns and strategies to overcome barriers to diabetes prevention and control.

**Discussion:**

This project demonstrated that it is feasible to screen for common health conditions in the supermarket setting and that combining screening with immediately accessible healthy food and educational resources can address multiple, intersecting barriers to diabetes prevention and management.

## 1 Introduction

Healthcare is distributed and experienced inequitably in the United States, contributing to disparities in morbidity, mortality, and the associated quality of life. Supermarkets, and specifically the pharmacies within them, located in regions underserved by healthcare systems may function as preventive care extension sites where screening and health promotion activities can occur. Residents of Health Professions Shortage Areas (HPSA), often also experience barriers to transportation, healthy food and physical activity (1–3). This contributes to disproportionately higher rates of diabetes, hypertension, and other metabolic disorders, and the difficulty controlling them (3–5). It is estimated that nearly 9 million people in the US have undiagnosed diabetes (which is 23% of those with diabetes) and 38% of the US population has prediabetes, though 80% are unaware that they have it (6). Left undiagnosed or undertreated, these health conditions, coupled with additional social and economic barriers, contribute to a cycle of chronic disease-related health deterioration, increased healthcare costs, and lower quality of life, particularly affecting adults as they age (3, 7–12). This urgent public health priority not only has implications for United States morbidity and mortality but also macroeconomic effects associated with strain on the healthcare system as well as individual household economic strain (3).

Population health strategies to improve preventive healthcare access include bringing care to familiar places like supermarkets and community centers (13, 14) growing the services provided by pharmacies, and addressing social needs affecting self-care—especially in a culturally responsive way (15–18). Many people see their pharmacist more often than their Primary Care Provider (PCP) for medication refills, guidance on over-the-counter medications and increasingly, vaccinations (19–21). Those who experience healthcare access barriers (transportation, co-pay costs) and/or have poor disease control can be reached in pharmacies (21–23). Interestingly, in New York state, people living in census tracts with high poverty, a lower percentage of residents with a college degree, and/or a high percentage of people identifying as Black/AA and/or Hispanic/Latine, have higher access to pharmacies (20).

Supermarkets (and their embedded pharmacies) have great potential as community-based sites of primary care extension, given that they are a source for medications and vaccinations as well as a hub for families to access food, household supplies, and connect with neighbors. Providing accessible, relevant, and consistent interventions within everyday spaces like supermarket pharmacies may help identify undiagnosed prediabetes and unmanaged diabetes, thus mitigating the health disparities experienced by these populations. Studies have demonstrated that supermarket interventions, inclusive of supermarket pharmacy interventions, have helped people make healthy food choices, provided screening for chronic health conditions, and assisted them with being adherent to prescribed medications (13, 14, 19, 24–28).

This project aimed to 1-determine the feasibility of screening for prediabetes and diabetes care in grocery stores with pharmacies located in urban areas, 2-identify unmet needs in healthcare access for people with diabetes, 3-provide education at the time of screening (evidence-based education material) and subsequently during interactive in-person education sessions on diabetes prevention and management, including healthy meal planning.

## 2 Context

The city of Buffalo, NY, where this project took place, is designated as a Medically Underserved Area (MUA) and Health Professions Shortage Area (HPSA). The East Side of Buffalo has a total population of 102,791. The demographic composition includes 61% African American, 16% White, 12% Asian, 10% Hispanic, and 1% identifying as Other. The median household income in this area is $35,648 ($21,790 per capita), and 36% of the population has an income below the poverty line (29). In the specific zip codes of interest, more than 30% of Black adults 65 and older have diabetes and more than half have hypertension, compared to 19% and 40% among White residents, respectively (30). Residents of these communities also experience insufficient number and access to food markets, known as food apartheid, resulting in food insecurity. To date, only one supermarket chain, Tops Markets, serves this region of Buffalo, which is largely populated by minoritized people due to historic and current structural racism including redlining and de facto segregation.

This project was born out of a partnership between Tops and researchers within the University at Buffalo (UB) Clinical and Translational Science Institute (CTSI). Additionally, we partnered with a pastor and parishioners of a local place of worship where two educational sessions took place. We termed the project SHARE – Supermarket Health Advocacy, Resources, and Education – and piloted the screening in four stores located in the city of Buffalo with support from a UB Civic Engagement Award.

## 3 Programmatic elements

### 3.1 Supermarket screening

Following IRB review and approval, a member of our UB CTSI Special Populations and Recruitment Team and one to two UB pharmacy student interns tabled at the entryway of four Tops Supermarkets between 10 am and 3 pm at Tops 25 times between January and June 2024. Sundays were the most effective recruitment days, with 146 (48%) participants screened. Patrons were invited to complete one of two questionnaires, depending on their reported diabetes status.

**Figure 1:**
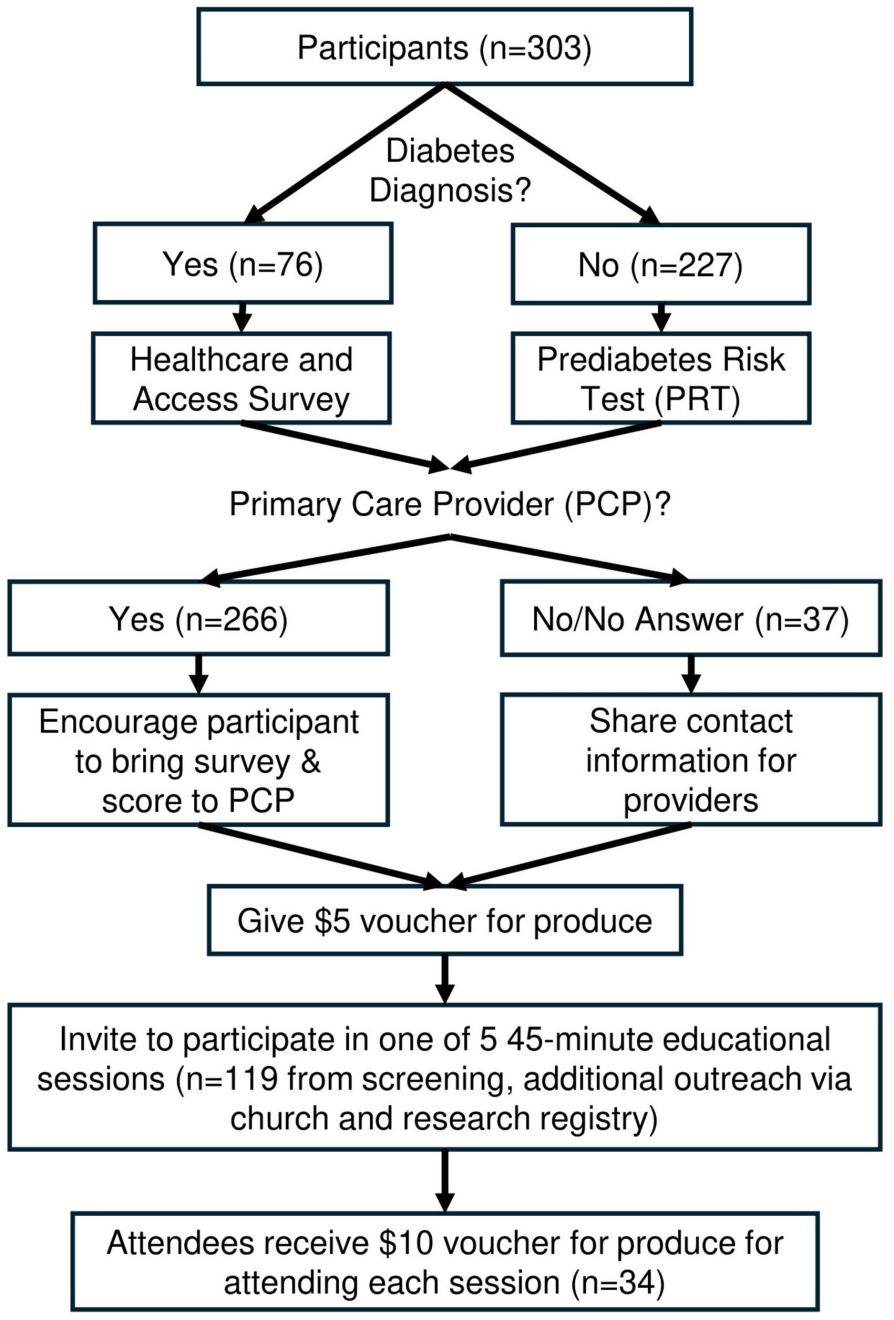
Prediabetes and diabetes healthcare screening and referral participant workflow.

If the participant stated they had not previously received a diagnosis of prediabetes/diabetes, they completed a Prediabetes Risk Test (PRT) (31). Validated in 2009 by Bang et al., this patient self-assessment tool has been endorsed by the Centers for Disease Control, American Diabetes Association and the National Diabetes Prevention Program (Table 1). If the participant had received a prior diabetes diagnosis, participants answered a diabetes questionnaire querying their diabetes knowledge and access to care. These questions, informed by evidence-based guidelines for diabetes management (32), included: (1) How was your Type 2 Diabetes (T2D) diagnosed? Check all that apply and please refer to our handout on Type 2 Diabetes and Pre-Diabetes. (2) Are you seeing your doctor every 3 months to monitor your diabetes? (3) Do you know what the importance of HbA1c is in monitoring your T2D? (4) Do you know your Body Mass Index (BMI)? (5) Is your blood pressure being checked at the doctor? (6) Is your cholesterol being checked at the doctor? (7) Are you having trouble paying for your medications or testing supplies? (8) Are you having problems with transportation to medical appointments? (9) Are you seeing a dentist every 6 months? (10) Are you seeing an eye doctor every year? (Table 2) The participant workflow following the PRT or the Healthcare and Access Survey is detailed in Figure 1.

**Table 1:** National diabetes prevention questionnaire results (*n* = 227).

	#	%
Age		
Younger than 40 (0)	60	26.4%
40–49 (1)	34	15.0%
50–59 (2)	47	20.7%
60 years or older (3)	86	37.9%
Sex		
Woman (0)	123	54.2%
Man (1)	104	45.8%
Ever Gestational Diabetes		
No/not applicable (0)	211	69.6%
Yes (1)	16	5.3%
Family with Diabetes		
No (0)	120	53.1%
Yes (1)	106	46.9%
Diagnosis of Hypertension		
No (0)	111	48.9%
Yes (1)	116	51.1%
Physically Active		
Yes (0)	163	72.1%
No (1)	63	27.9%
Weight Category		
Not at risk (0)	58	25.6%
Column 1 (1)	86	37.9%
Column 2 (2)	59	26.0%
Column 3 (3)	24	10.6%
Total Score 5 or Higher		
No	95	41.9%
Yes	132	58.1%

Numbers in parentheses correspond to the Risk Scoring Protocol.

**Table 2:** Healthcare use, diabetes health literacy and social barriers among people reporting diabetes diagnosis (*n* = 76).

Question		#	%
Healthcare Use	Are you seeing your doctor every 3 months to monitor your diabetes?	68	90.7%
	Is your blood pressure being checked at the doctor?	76	100.0%
	Is your cholesterol being checked at the doctor?	75	98.7%
	Are you seeing a dentist every 6 months?	47	61.8%
	Are you seeing an eye doctor every year?	66	86.8%
Diabetes Health Literacy	Do you know what the importance of HbA1c is in monitoring your T2D?	55	72.4%
	Do you know your Body Mass Index (BMI)?	38	50.0%
Social Barriers	Are you having trouble paying for your medications or testing supplies?	14	18.4%
	Are you having problems with transportation to medical appointments?	11	14.5%

After assuring participants of confidentiality, we gave them the option to share contact information in order to receive updates on five educational sessions to take place in June and July 2024. Participant contact data and responses to the questionnaire were entered in a password protected Excel file and deidentified for analyses. Analyses included descriptive statistics, including frequencies and cross-tabulations with chi-square tests.

### 3.2 Community educational sessions

Five educational sessions were scheduled in early evenings at Hopewell Baptist Church and Merriweather Library—both located on the east side of Buffalo. The sessions were advertised using the contact information participants had previously provided as well as invitations sent out through the UB CTSI Buffalo Research Registry (which includes Western New York community members who are interested in participating in research) and by word of mouth to parishioners of the partnering church.

Sessions used effective education strategies, with facilitators and participants seated in a circle as equals (32). This approach recognizes that knowledge, in this case related to prevention and control of diabetes, does not just come from the healthcare system experts, but can also emerge from groups sharing their life experiences and management strategies (33). The CTSI Recruitment and Special Populations co-directors, a medical anthropologist/health services researcher and a pediatric endocrinologist, were the lead facilitators. CTSI staff supported the sessions through recruitment, set-up, water and healthy snacks procurement, voucher distribution, note-taking, and active participation. Using flipchart and an easel, the lead facilitator (Cadzow) documented the discussion prompts as well as participant responses throughout the session. Light refreshments were provided.

Session 1 focused on perceptions of living with diabetes and strategies for health behavior change. Session 2 discussed pre-planning strategies (e.g., grocery lists, meal prep). Session 3 included an overview of the physiology of diabetes and barriers to behavior change. Session 4 provided an overview of prevention strategies both at the individual and community levels. Session 5 focused on strategies to improve communication with doctors. Sessions began with introductions and check-in and participants sharing their expectations followed by interactive activities to initiate group participation in conversation. These included:

*Living survey:* We posed a statement to which participants indicate they “agree,” “disagree,” or feel “neutral/not sure” about. These statements align with common beliefs about diabetes. Respondents were encouraged to indicate their first reaction to the statement. Statements used for this exercise included: (1) You can have diabetes and be a healthy person. (2) My community has plenty of resources to support people who have prediabetes or diabetes. (3) People with diabetes can still eat a variety of foods, including foods with added sugar. (4) It is very difficult to change your health behaviors when you learn that you are at risk for diabetes.

*Smaller groups break outs focused on:* brainstorming strategies to achieve the goals determined by participants, such as cutting 200 calories per week, preplanning physical activity, procuring, preparing and cooking healthy food, and discussing prevention strategies in the categories “what can you do on your own?” “what can you do with some help?” and “what is someone else’s job, and what resources need to be there?” The breakout sessions were followed by participants reporting strategies back to the full group.

Finally, the sessions ended with a round-robin check-out, which included the prompts, “how are you?” “what did you like and/or learn?” “what will you take back and apply?” and “what should we change or do differently in future sessions?” At the session’s end, participants were given $10 Tops supermarket vouchers and invited to attend a future session. Meeting notes were analyzed for key themes using a general inductive approach (34).

## 4 Results

### 4.1 Demographics

Over 6 months, a total of 303 participated in the project at four Tops grocery stores, with most (n = 257) screened at two locations (Jefferson and University Plaza). About 54% were female and close to 40% were age 60 and older.

### 4.2 Survey findings

Out of the 303 participants, 76 (25%) reported a diabetes diagnosis and 227 (75%) did not. Of the 227, 58% scored 5 or higher on the Prediabetes Risk Test, indicating that they were at risk for prediabetes. 75% were overweight or obese (based on calculated BMI with self-reported weight and height). Not surprisingly, they experienced additional co-morbidities including hypertension (51%). Nearly half had a family history of diabetes (Table 1). Among those who reported a diabetes diagnosis, most people regularly saw their doctor, however there were lower numbers who saw their dentist and understood HbA1c and BMI. There were also social barriers for several respondents (Table 2).

Among all participants, 287/303 answered they had a PCP. However, the percentage among people not at-risk for diabetes who were without a PCP was 14% compared to only 4% of those who screened at risk for diabetes. Only 3% of people who reported having been diagnosed with diabetes did not have a PCP. This difference by diabetes status was statistically significant (*p* = 0.005).

Healthcare utilization among people with diabetes was related to social barriers (income and transportation) as well as diabetes-related health literacy. Compared to those who saw a dentist every 6 months, those who did not see a dentist had more trouble paying for medications or testing supplies (14.9% vs. 24.1%) and problems with transportation to medical appointments (10.6% vs. 20.7%). There were statistically significant differences between those who did and did not see an eye doctor yearly. Those who did not see an eye doctor regularly had more payment troubles (12.1% vs. 60.0%, *p* = 0.002) and transportation problems (7.6% vs. 60.0%, *p* < 0.001).

The association between healthcare utilization and diabetes health literacy was most observable related to the knowledge of the importance of HbA1c. People with diabetes who did not see the dentist every 6 months were more than twice as likely to not know the importance of HbA1c (44.8% vs. 17.0%, *p* = 0.009; Table 2). Those who did not see the eye doctor yearly were more than 2.5 times more likely to not know the importance of HbA1c (60.0% vs. 22.7%, *p* = 0.023; Table 3). Findings trended in this direction for knowledge of BMI related to seeing an eye doctor.

**Table 3:** Healthcare utilization by diabetes health literacy (knowledge of BMI and HBA1c; *n* = 76).

Healthcare utilization		Answered “No” to “Do you know your Body Mass Index?”			Answered “No” to “Do you know what the importance of HbA1c is in monitoring your health?”		
		%	#	*p*-value	%	#	*p*-value
Are you seeing a dentist every 6 months?	No (*n* = 29)	58.6%	17	0.173^a^	44.8%	13	0.009**,^b^
	Yes (*n* = 47)	44.7%	21		17.0%	8	
Are you seeing an eye doctor every year?	No (*n* = 10)	30.0%	3	0.115^c^	60.0%	6	0.023*,^d^
	Yes (*n* = 66)	53.0%	35		22.7%	15	

a *χ^2^* = 1.394, *df* = 1.

b *χ^2^* = 6.934, *df* = 1.

c *χ^2^* = 1.842, *df* = 1.

d *χ^2^* = 6.033, *df* = 1.

*p*-values reflect Fisher’s Exact Test significance (1-sided). Numbers in parentheses indicate row percentages.

Results demonstrate the ability to conduct supermarket screening for diabetes risk and social barriers as well as referral to healthcare resources. Regarding the potential to further engage participants in program offerings, we reviewed the participants who provided their contact information, who signed up for a community-based educational session, and who attended a session. Over a third of participants who had a PCP shared their contact information (100/266, 37%) and half of those without a PCP shared their contact information (19/37, 51%). All 119 participants were contacted via their preferred method (email, text, phone call) for diabetes education sessions. Ten people signed up for a session and one attended. The 33 other attendees learned about the sessions through other efforts (church, Buffalo Research Registry). Table 4 shows the breakdown by type of outreach.

**Table 4:** Intended and actual attendance resulting from outreach to screening participants.

	Total contacted	Responded/ reached	Committed to or signed up to attend	Attended
Outreach details	#	#	#	#
Contacted by email/text	56	5	2	1
Contacted by phone call	52	16	8	0
Error or missing contact information	12	–	–	–
Total	119	21	9	1

### 4.3 Educational session outcomes

Thirty-four people attended one or more community educational sessions held in a local church and library. The two church sessions had the highest attendance. Fifteen participants attended more than one session; several shared that they valued the gatherings and wished that they could continue. They functioned not just as educational opportunities but also as supportive environments to reveal and discuss fears, concerns and successes in their health journeys.

The education session agenda informed the overarching categories identified in qualitative analyses. These categories are: Reason for Attendance/Expectations/Questions, Perceptions of Diabetes, Barriers and Facilitators to Self-Management/Prevention of Diabetes, and Topics of Future Interest.

At the start of the session, the lead facilitator welcomed participants and invited them to share their name, how they were doing (scale of 1–5) and why they attended the session. Many participants also shared their diabetes status.

Respondents said that they generally wanted to learn more to help themselves or loved ones. Specific questions related to: (1) The physiology of diabetes (what is it, different types, how you get it, the risk factors, the warning signs, what happens when your blood sugar fluctuates, diabetic coma). (2) Health behaviors that prevent and/or control/manage diabetes (monitoring blood sugar, second opinions, the role of the endocrinologist, healthy food choices, eating in moderation and realistic expectations, and how living in a food desert (words used by the participants) impacts food choices and diabetes risk). (3) Medicines for diabetes control/management (Ozempic, Jardiance), finding the right medicine, and the ability to wean off medicine with health behavior change.

Group perceptions about diabetes and associated resources in the region were elicited through the “agree/disagree/neutral” activities in the first three community educational sessions. The following statements are followed by the feedback shared by participants.

*You can have diabetes and be a healthy person*. Those who agreed to this statement felt that if you have it controlled through healthy diet and exercise, you can be healthy. “Everyone has something. It is normal to have something going on. You can still be healthy.” Those who disagreed said that “you always have to worry, pay attention,” saying that you are always watching whether your sugar goes up or down or if you do not feel well. They felt that when you have diabetes, you have an illness, therefore, you are not healthy.

*My community has plenty of resources to support people who have prediabetes or diabetes*. A couple of people agreed, citing specific resources available through a medical provider and insurance company. Some felt neutral, as they had been given referrals for resources but just had not made any appointments yet. Most participants disagreed, stating that there are not enough resources for healthy food, that available resources are costly, insurance often does not cover resources and sometimes doctors do not clearly tell you that you have or are at risk for diabetes. They also stated that there is limited-to-no access to specialists and when they can access them, specialists are unresponsive to their needs, do not listen, and recommend drastic lifestyle changes that are often not attainable. Participants also indicated that many people do not know how to change diet or lifestyle.

*People with diabetes can still eat a* var*iety of foods, including foods with added sugar*. About half of the group to whom this statement was posed agreed that one “can eat whatever you want in moderation.” The other half disagreed with the statement, stating that when you have diabetes, you really need to avoid sugar. Some discussion ensued around the pros and cons of artificial sweeteners as an alternative.

*It is very difficult to change your health behaviors when you learn that you are at risk for diabetes*. Among those who agreed with this statement, responses included “you got used to living your life one way, and now you have to stop and establish a new behavior and routine.” One person shared that when the baseball game is on at 10 pm, he eats a lot of midnight snacks. People said that healthy food is expensive, your body craves sugar, and that it helps to have an advocate or accountability partner. Some said that they know people who have the mentality that “they will die anyway” and should enjoy the foods they like in the present. Respondents who disagreed to this statement shared the sentiment that “we all need to take care of our bodies as this is the only body we get.” One person felt terrified after seeing family members “go down the long medical road with diabetes,” so she started exercising more after her diagnosis.

Participants shared their barriers to prevent and control diabetes, including difficulties controlling cravings or stimuli, lack of social support, time constraints, the cost of healthy food, and environmental triggers. Participants in all sessions discussed strategies that would help them prevent and/or control diabetes. This included enjoyable ways to stay active, shopping habits (using lists, reading labels), habits related to meals and snacking (portion sizes, avoiding processed foods, tracking calories, avoiding late night snacking/eating, healthy substitutions, gradually decreasing sugar), and cooking approaches (baking vs. frying, weekly meal preparation). They also discussed medication management, including wearable sensors/monitors to avoid frequent finger sticks, and talking with their doctor.

The last session included a discussion about talking with physicians, unfortunately highlighting the often-inadequate relationship that exists between doctor and patient. Respondents shared that they had experienced gender, racial, economic and age bias from healthcare providers. One respondent said that a doctor can be “bigheaded,” only looking at the numbers and not the person. Some doctors are “just looking for a quick fix (e.g., pill) and do not want to get to the root of the problem.” One participant encouraged folks to share information about themselves and their family history and to remember that “you know your body best.” In general, participants reiterated that better communication is needed between doctors and patients.

Participants had recommendations related to what they would like learn more about and what would improve diabetes-related care in their communities. Participants wanted to learn more about how to pre-plan meals, what types of medication interventions are available, the role of genetics in predicting diabetes risk, what resources are available in their communities (e.g., endocrinologists), and how to cook foods that are healthier but still taste good (e.g., cooking classes).

Participants shared several strategies that would result in improvements to healthcare experiences for people with diabetes. These included: creating better technology for blood sugar monitoring, increasing insurance coverage to cover diabetic assistive products, focusing on preventive care rather than post-diagnosis management, readily available and clear explanations of insurance benefits, mobile wellness vans, and universities conducting research and advocacy in community settings to gather and feature the voice of those most affected.

## 5 Discussion

Screening for prediabetes risk and diabetes management in the supermarket once weekly over 6 months identified 132 people who were at risk for prediabetes as well as gaps in health literacy (HbA1c), healthcare use (dentist), and social barriers (income and transportation) among those with a diabetes diagnosis. Providing an efficient screening process with $5 food vouchers was an effective way to engage people in a familiar and frequented community setting. Of note, 39% of the screened participants volunteered contact information for follow-up, particularly among people who scored at-risk for prediabetes or self-disclosed a diabetes diagnosis (45%). This facilitated outreach for community educational sessions and additional diabetes health-related resources is promising for future supermarket-based screenings that are structured to include follow-up.

Studies have found that perceived severity (e.g., experiencing disease symptoms) is associated with healthcare seeking behavior (34). In our project, those who were not at risk for prediabetes or currently diagnosed with diabetes were less likely to have a PCP. This supports previous research that shows the absence of perceived risk, associated with having symptoms or diagnosis of a chronic condition, relates to lower use of preventive healthcare (34). This potentially leads to later diagnosis and treatment for emerging chronic conditions. Of the 227 who took the PRT, 58% had a score indicating prediabetes risk. The national rate of prediabetes is 38%, with the majority (80%) unaware of their status. The higher rate in our group could indicate that the populations in these regions have higher risk for prediabetes than the national average, driving home the importance of frequent community-based screening. We cannot rule out that people with higher perceived risk and/or severity were more likely to approach the table to be screened. In either case, risk was identified among a population who were otherwise not aware of having and/or being at risk for developing diabetes. Also, people who perceive themselves as “healthy” are less willing to participate in health screenings due to lack of time, belief that the screening is too complicated, negative emotions like fear or discomfort, or a negative healthcare experience (35). The finding that 95 participants (42% of the 227 who completed the PRT) had scores that did not indicate risk suggests that the location, time required, and approach appealed to at least some shoppers who were moderately more healthy.

Among respondents who reported a diabetes diagnosis, primary healthcare use was generally consistent with the standard of care in diabetes (91% saw their doctor every 3 months). However, this population still presented gaps in healthcare access, health literacy and diabetes knowledge. As reported above, 38% did not see the dentist every 6 months and 28% did not know the importance of HbA1c in monitoring their diabetes. Consistent with other studies of adults with diabetes, this has important implications for future interventions (36–39). Given that people with diabetes have 2 or 3 times the rate of periodontal disease, compared to those without diabetes, regular dental visits are critical to managing dental health and preventing additional complications that could impact eating ability as well as other chronic disease conditions like heart disease (37). It is not surprising that people who understand the significance of HbA1c report better diabetes care and therefore fewer diabetes complications (39–41).

About 18% of our respondents struggled to afford medications, and about one out of six had transportation issues, highlighting gaps that enhanced primary care access and healthcare resources in the community can address. Medication affordability impacts adherence (42). Working with people to access financial assistance will improve their ability to manage their diabetes. Nationally, it is estimated that more than one in five adults who have limited access to public transit forgo healthcare due to transportation barriers (43). Positioning healthcare screening in community spaces and expanding the role of the supermarket pharmacy in chronic disease management may help to partially mitigate these transportation barriers.

Finally, related to education, providing group diabetes education is well established as effective (44). Given the pilot nature of this project and the expectation that participants may only attend one session, we opted not to implement a structured National Diabetes Prevention Program or ADCES Diabetes Care and Education Curriculum, but to rather have participants co-produce the topics to be covered during each session, using a popular education approach. The content shared by facilitators during these sessions, though, was evidence-based from the National Diabetes Prevention Program. As the sessions were open to all, those who attended ranged from people who were just diagnosed with prediabetes or diabetes and experiencing difficulty with lifestyle changes to people with well-controlled diabetes as well as caregivers of people with diabetes (44). This was an ideal composition to support the popular education strategy of teaching and learning together. In general, participants appreciated the open conversation and collective sharing of strategies for management. Those who had managed diabetes successfully for many years shared their strategies with people who were newly diagnosed and/or were not effectively managing their condition. Comfortable with the community setting, many attendees expressed interest in ongoing meetings to share their journeys or those of their loved ones as they manage or attempt to prevent diabetes.

These results suggest that future community-based interventions can place screening, care, and education in accessible, familiar locations to reduce (but not eliminate) barriers like health insurance and transportation. Using popular education fosters empowerment and shows promise in promoting health behavior change through culturally-responsive strategies and strengthened social connections (45). There are future opportunities to blend popular education strategies with structured evidence-based curriculum for comparability with other intervention programs. Utilizing non-traditional settings like grocery stores and pharmacies can enhance engagement with underserved populations and improve early detection and intervention. This model of using validated screening instruments along with referral and education in supermarket settings has been or can be applied beyond the scope of diabetes care to address other chronic conditions (mental health, cancer, heart disease/hypertension, dental care, etc.) (46–49). Further, in under-resourced regions, there is a need for additional resources to address transportation and medication expenses as well as social support for resource navigation, tailored guidance, and ongoing support like that provided by Community Health Workers in order to prevent chronic conditions and improve self-management (3, 5, 7).

### 5.1 Acknowledgment of conceptual or methodological constraints

Despite the 39% of screened participants willing to share their contact information, the majority did not attend the education sessions; these were largely attended by other community outreach. A few factors may explain this. We did not provide dates, times, and locations of the education sessions at the time of the supermarket screening. This was a lost opportunity to alert potentially interested shoppers about this community resource at the first point of contact. Also, we learned that we must diversify the locations for the education sessions (they were close to one of the Tops supermarkets but not to the other three). In the future, we will identify community settings close to each of the participating supermarkets and will schedule the sessions well in advance, to share with patrons at the point of screening.

As this project was conducted primarily to determine feasibility of reaching people through the supermarket, we did not collect additional participant demographic data that might contribute to our understanding of their barriers and facilitators (e.g., education, income, the zip code of residence). Low income and low education are associated with higher risk for obesity and lower adherence to healthy diets defined by USDA’s Dietary Guidelines. This contributes to risks related to prediabetes as well as diabetes management (50). Also, we did not ask about healthcare access behaviors among the participants who completed the PRT questionnaire, given that we were trying to limit the “ask” of people entering the supermarket to a short survey. It would be interesting to know the behaviors of people without a diabetes diagnosis related to dental and eye care as well as barriers like transportation and medication cost as we consider the application of this approach to prevention as well as management of diabetes. We also did not measure the impact of supermarket screening on subsequent health behaviors. We do not know whether those who screened at-risk for prediabetes made inquiries or appointments with their doctor. We also did not measure whether those attending the education sessions made any lifestyle changes.

### 5.2 Resource identification initiative

To take part in the Resource Identification Initiative, please use the corresponding catalog number and RRID in your current manuscript. For more information about the project and for steps on how to search for an RRID, please click here.

## Data Availability

The raw data supporting the conclusions of this article will be made available by the authors, without undue reservation.
